# *IDH* mutation and *MGMT* methylation status in glioblastoma and other gliomas patients: a Russian retrospective cohort study

**DOI:** 10.1186/s43046-025-00296-w

**Published:** 2025-05-16

**Authors:** Moez Eid, Dema Alset, Nataliya Timoshkina, Dmitriy Gvaldin, Eduard Rostorguev, Sergey Kavitskiy, Inna Novikova

**Affiliations:** National Medical Research Centre for Oncology, Rostov-on-Don, Russian Federation

**Keywords:** Glioma, *IDH*, *MGMT*, Survival rate, Glioblastoma

## Abstract

Glioma is a devastating type of brain tumor with high malignancy, an extremely high mortality rate, and a recurrence risk. Molecular markers are known to have a major role in classification, prognosis, survival rate, and therapy determination for different glioma subtypes. The aim of this study was to investigate the association of gliomas’ main genetic markers: isocitrate dehydrogenase (*IDH*) mutations and O6-methylguanine-DNA methyltransferase (*MGMT*) promoter methylation status with the survival rate in Russian patients with glioblastoma and other glial tumors. According to histological subtype, included glioma patients were divided into two groups: glioblastoma (*n* = 90) and other gliomas (*n* = 40). *IDH* mutations were screened by high-resolution melting-curve analysis (HRM) followed by direct sequencing, and *MGMT* methylation was detected with pyrosequencing. Our data showed that *IDH* mutations are significantly more frequent among patients with other gliomas compared to glioblastoma patients (*p* < 0.001). Patients with mutated *IDH* gene have a significantly higher progression-free survival (PFS) and overall survival (OS) rates than those with wild-type genes. *MGMT* promoter methylation status was found to be significantly associated with PFS, but not OS. The presence of *IDH* mutation with a methylated *MGMT* promoter significantly increased patients’ PFS and OS. To our knowledge, this is the first study to investigate the association of *IDH* and *MGMT* genetic biomarkers with glioma in the Russian population. Our findings could be used in future studies to improve glioma prognosis and classification and reach a personalized treatment protocols depending on multiple molecular biomarkers.

## Introduction

Gliomas are the most abundant histological type of central nervous system (CNS) tumors, accounting for 27% of all neural malignancies and 80% of neural primary malignant tumors [21]. They arise from different types of glial cells and have different incidences in each country. Worldwide, about 100,000 adults per year are diagnosed with diffuse glioma [16]. In the Russian Federation, according to 2019 statistics, 10–13/100,000 per year are glioma patients [24].

The most critical point in gliomas individualized treatment is accurate classification. From 2007 until 2016, this group of brain malignancies was classified based on the histological features into high grade (IV: glioblastoma, the most common and aggressive subtype, accounts for 49% of malignant brain tumors) and low-grade (II–III: astrocytoma and oligodendroglioma in about 30% of brain malignancies) [20, 21]. In 2016, the WHO published a new classification system integrating morphological and molecular features of glioma (genetic mutations mainly). The classification of CNS tumors in 2022 was the most detailed [16].

Diagnostic and prognostic molecular markers are known to have a major role in gliomas. This mainly concerns isocitrate dehydrogenase (IDH), the main diagnostic genetic marker for glial tumors. *IDH* encodes enzymes converting isocitrate to alpha-ketoglutarate (*α*-KG) using nicotinamide adenine dinucleotide phosphate (NADP^+^) as a cofactor to produce NADPH, one of the most important antioxidants [11]. *IDH* mutations cause the enzyme to have a neomorphic function converting *α*-KG into D-2-hydroxyglutarate (2-HG), one of the known onco-metabolites [12]. 2-HG inhibits the functions of *α*-KG-dependent dioxygenases, resulting in DNA and histones hypermethylation and thus tumorigenesis [23]. It is already known that glioblastoma *IDH* wild-type patients have poor prognosis. However, the exact effect of *IDH* mutations on survival rate is not fully understood [9].

*MGMT*, which encodes the DNA repair protein O6-methylguanine-DNA methyltransferase, is located on chromosome 10q26.3, containing 98 CG-rich dinucleotide sequences (CpG-islands). The methylation status of these CpGs is known to modulate genetic expression, and thus, it was previously studied in several tumors. Furthermore, *MGMT* methylation status is proved to be a prognostic factor for chemotherapeutic response to alkylating agents such as temozolomide [13]. It is known that temozolomide inhibits DNA replication of tumor cells through the alkylation of guanine at the O6 position. MGMT removes the alkyl groups from guanine at the same position, thereby counteracting the effect of temozolomide. Methylation of the *MGMT* gene promoter silences genetic expression, leading to a reduction in MGMT levels and improving the response to alkylating agents [7].

The aim of current research is to study the association of gliomas main genetic markers: *IDH* mutations and *MGMT* methylation status with survival rate in Russian patients with glioblastoma and other glial tumors. In addition, we aimed to confirm the association of these markers with each other’s and with glial subtype.

## Materials and methods

### Study subjects

Between December 2018 and December 2021, 131 glioma patients were sent to the laboratory of molecular oncology in the National Medical Research Centre for Oncology (Rostov-on-Don, Russian Federation) and were retrospectively recruited in the study. One patient with missing information on *IDH* status was excluded. Morphological and immunohistochemical analyses were performed to confirm the diagnosis and identify the histopathological subtype of glioma for every enrolled patient. Included participants were classified into two groups: glioblastoma (*n* = 90) and other gliomas (*n* = 40), including anaplastic astrocytoma (*n* = 21), diffuse astrocytoma (*n* = 14), oligoastrocytoma (*n* = 2), and dendro-glioma (*n* = 3). All participants have willingly signed informed consent before their recruitment. The study had the approval of the Ethical Committee of the National Medical Research Centre for Oncology (protocol no. 6) and was conducted in accordance with the Declaration of Helsinki.

### DNA extraction

Genomic DNA was isolated from formalin-fixed paraffin-embedded cancer tissue blocks using the MagPure FFPE DNA LQ kit B (Magen, Beijing, China). DNA concentration was measured fluorometrically using the Qubit 2.0 (Life Technologies, USA). All DNA samples with sufficient concentration were stored at − 20 °C until the start of the study.

### Detection of IDH1 and IDH2 mutations

A fragment of exon 4 of *IDH1*, spanning the R132 mutations hotspot of *IDH1*, and *IDH2* entire exon 4 of IDH2 were amplified from genomic DNA by polymerase chain reaction (PCR), and mutations were screened by high-resolution melting-curve analysis (HRM) followed by direct sequencing.

The DNA concentration was adjusted to 12.5 ng/μl. The primer sequences used for PCR were as follows: forward, 5′-TCT TCA GAG AAG CCA TTA TC-3′ and reverse, 5′-CAC ATT ATT GCC AAC ATG A-3′ for *IDH1* (amplicon size 119 bp) and forward, 5′-AAA CAT CCC ACG CCT AGT CC-3′ and reverse, 5′-AAA GTC TGT GGC CTT GTA CTG C-3′ for *IDH2* (amplicon size 172 bp). PCR reactions were performed in duplicate in a 25-μl reaction mixture containing the following: 14.28-μl nuclease-free water, 2.5 μl 10 × PCR buffer (20-mM MgCl_2_) (Roche Diagnostics, Meylan, France), 0.4-μl dNTPs (12.5 mM), 0.5-μl MgCl_2_, 1.2-μl forward primer (10 μM), 0.12-μl reverse primer (10 μM), 0.5-μl TaqM buffer (10 μM), 0.5-μl Taq-polymerase (5 U/μl), and 5 μl of isolated DNA.

**Fig. 1:**
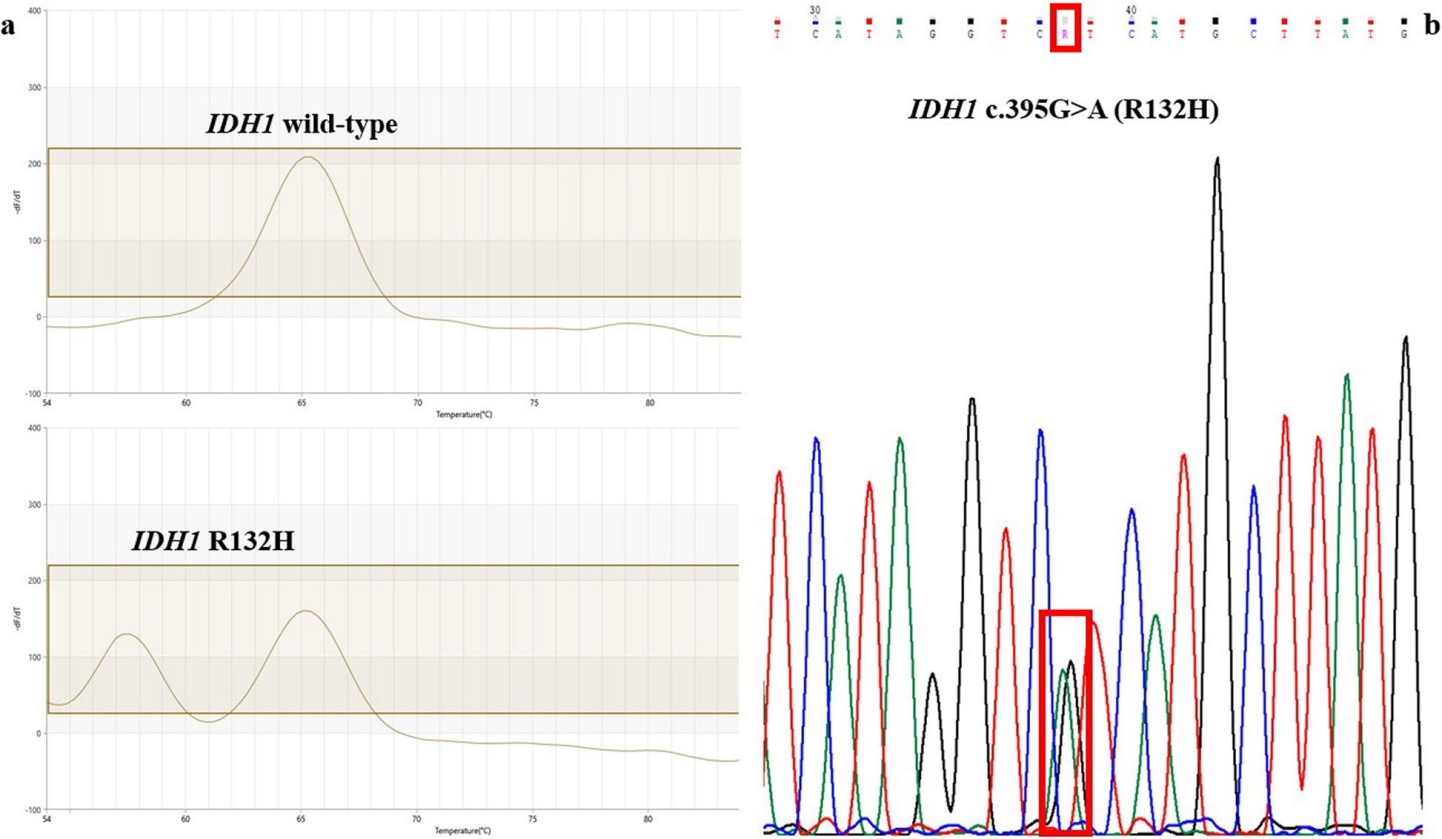
Detection of *IDH1* R132H mutation by **a** HRM analysis and **b** Sanger sequencing

PCR and HRM analyses were performed in a single run using the LightCycler 480 instrument (Roche Diagnostics, Meylan, France). The cycling conditions were as follows: an initial denaturation of 3 min at 95 °C, followed by 40 cycles of amplification consisting of denaturation at 95 °C for 13 s, annealing at 54 °C for 40 s, and extension at 72 °C for 20 s. HRM is based on computer analysis of DNA melting transitions. Post-amplification HRM was performed as follows: 1 min at 95 °C, 8 min at 54 °C, followed by gradual heating of the samples at a rate of 0.3 °C/12 s from 54 to 90 °C. PCR with the same protocol was also performed for positive control of each studied mutation and negative control (nuclease-free water instead of isolated DNA). LightCycler 480 software release 1.5.0 SP4 (Roche Diagnostics, Meylan, France) was used for all data analysis including fluorescent melting curves. An example of HRM results is shown in Fig. 1a.

After HRM, samples were purified and sequenced bidirectionally, using the same primers and the BigDye Terminator v1.1 sequencing kit (Applied Biosystems, Thermo Fisher, USA). All sequences were analyzed for somatic mutations using seqscape software (Applied Biosystems, Thermo Fisher, USA). Figure 1b represents an example of a sequencing result.

### Bisulfite conversion and MGMT promotor methylation pyrosequencing analysis

Bisulfite conversion of up to 250 ng of isolated DNA was performed using the EpiJET Bisulfite Conversion Kit no. K1461 (Thermo Fisher Scientific, USA) according to the manufacturer’s instructions. A duplicate of each sample, universal polymethylated DNA, and unmethylated DNA (positive and negative controls) were also subjected to bisulfite conversion. All samples with bisulfate conversion efficiency of less than 95% were excluded.

**Fig. 2:**
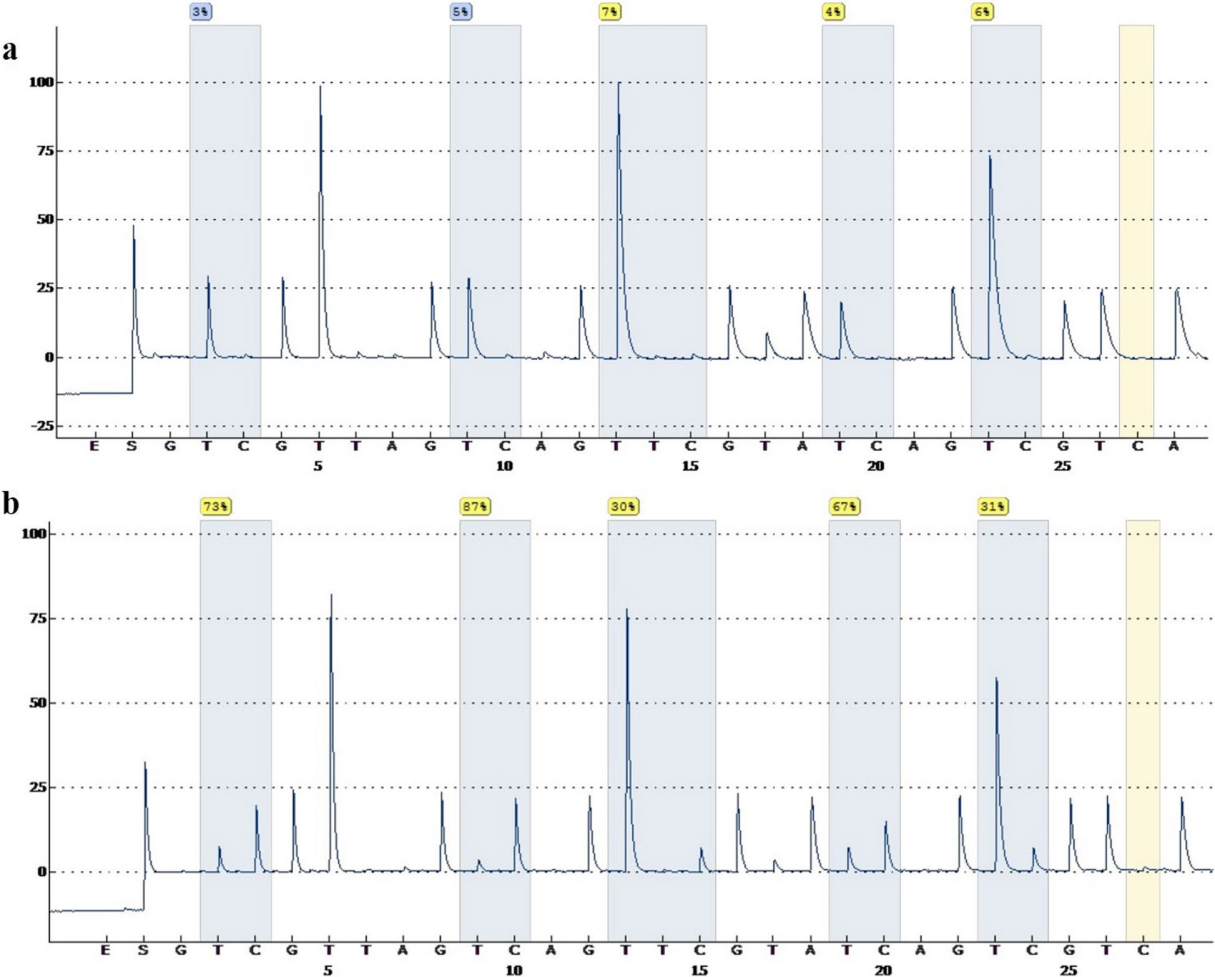
Detection of *MGMT* methylation by pyrosequencing. Pyrograms demonstrate **a** unmethylated and **b** hypermethylated CpG islands

Pyrosequencing analysis was carried out for 5 CpG sites in exon 1 (positions 17 to 39, Ensembl ID: OTTHUMT00000051009) of the *MGMT* promoter with the PyroMark Q24 CpG MGMT kit, according to the manufacturer’s recommendation. PCR was performed with 1-μl bisulfite-treated DNA, 0.5 μl (5 pmol) of each primer, 1 × PCR buffer, 1.5-mM MgCl_2_, 0.2-mM each dNTP, 0.8 U Taq polymerase (provided in the kit), and MilliQ water to 25 μl. PCR conditions were 95 °C for 15 min; 45 cycles of 95 °C for 20 s, 53 °C for 20 s, 72 °C for 20 s, and 72 °C for 5 min. PCR products of 104 bp were checked by 2% agarose gel electrophoresis. Subsequent quantification of the methylation density was performed using the PyroMark Q24 software. Examples of results for unmethylated and hypermethylated *MGMT* promoters are shown in Fig. 2a and b, respectively. When the methylation percentage was 10% or higher, the sample was classified as methylated.

### Statistical analysis

Statistical analysis was performed using SPSS Version 27 (SPSS IBM Corp., Armonk, NY, USA) with *p*-values ≤ 0.05 considered statistically significant. Continuous clinical characteristics are presented as mean ± standard deviation. The Student *t*-test was used to compare continuous variables. To assess the differences in *IDH* mutations between studied groups, the chi-square test was performed, and odds ratios (OR) with 95% confidence intervals (CI) were calculated. Progression-free survival (PFS) was defined as the time between first-line treatment and the incidence of first relapse. PFS and overall survival times were analyzed by the Kaplan–Meier method and stratified log-rank test. Hazard ratios (HR) and 95% confidence intervals (95 CI) were calculated.

## Results

### Patients’ characteristics

One-hundred thirty glioma patients aged 9–74 years old (mean ± SD: 54.15 ± 13.58) were included in the study. Clinical characteristics of them are presented in Table 1, including age, sex, PFS, overall survival, *IDH* mutational status, and *MGMT* promotor methylation status. The male/female ratio was the same in the studied two groups (*p* = 1.00). A significant difference by age was detected between glioblastoma and other glioma patients (*p* < 0.001). In addition, PFS was significantly different between glioblastoma and other glioma patients (*p* = 0.002). The majority of glioblastoma patients had a PFS of less than 6 months (56.7%). Patients with a PFS of more than 2 years were more common in other gliomas, in comparison with glioblastoma (15% and 2.2%, respectively). It should be mentioned that first-line treatment for all patients was maximal surgical resection. In most cases, surgery was followed by radiotherapy and chemotherapy.

**Table 1:** The characteristics of Russian glioma patients included in the study

	**Glioblastoma (*n* = 90)**	**Other gliomas (*n* = 40)**	***p*-value**
Age at diagnosis (mean ± SD)	57.96 ± 11.3	45.6 ± 14.6	< 0.001*
Age groups (years)			
< 40	8	16	< 0.001*
40–60	35	16	
> 60	47	8	
Sex			
Male	50	22	1.00
Female	40	18	
PFS (months)			
< 6	51	12	0.002*
6–12	19	8	
13–24	14	12	
> 24	2	6	
Missing**	4	2	
Overall survival (months)			
< 6	15	2	< 0.001*
6–12	25	4	
13–24	30	5	
> 24	20	27	
Missing**	0	2	
*IDH* mutational status			
Wild type	84	16	< 0.001*
IDH1 (R132H)	6	22	
IDH1 (R132G)	0	1	
IDH2 (R172 W)	0	1	
MGMT promotor methylation status (%)			
< 10	36	11	0.60
10–30	21	10	
> 30	19	9	
Missing**	14	10	

^*^p-value < 0.05 (significant variation). **Survival data and *MGMT* promotor methylation status for some patients are missing

### The correlation of IDH mutation with MGMT methylation status in gliomas

Among all included patients, 30 had a mutated *IDH* gene (23.07%). Evaluating the correlation of these mutations with *MGMT* promoter methylation status, we found that methylated promoters were more frequent in patients with *IDH* mutations (72%), compared to those with the wild-type variant (50.6%). However, this correlation was statistically insignificant. The *p*-value and odds ratios are shown in Table 2.

**Table 2:** The association of *IDH* mutation with *MGMT* promoter methylation status in glioma patients

	***IDH* mutated**	***IDH* wild type**	**Chi-square (*p*-value)**	**OR (95% *CI*)**
*MGMT* unmethylated	7	40	3.54 (0.06)	0.49 (0.15–1.06)
*MGMT* methylated	18	41		2.51 (0.95–6.66)

### IDH mutations and MGMT promoter methylation in glioblastoma and other gliomas

Our data showed that *IDH* mutations are significantly more frequent among patients with other gliomas compared to glioblastoma patients (*p* < 0.001). Furthermore, the majority of glioblastoma patients were carriers of the *IDH* wild-type variant (*OR* = 2.33, 95% *CI* = [1.59–3.42]). No significant association was noted between *MGMT* promoter methylation status and glioma subtype. Results are presented in Table 3.

**Table 3:** The association of *IDH* mutation and *MGMT* promotor methylation with glioma subtype

	Total	Glioblastoma	Other gliomas	Chi-square (*p*-value)	OR (95% *CI*)
IDH					
Wild type	100	84	16	44.37 (< 0.001*)	2.33 (1.59–3.42)
Mutated	30	6	24		0.11 (0.04–0.25)
MGMT promoter					
Methylated	59	40	19	0.998 (0.31)	0.64 (0.27–1.53)
Unmethylated	47	36	11		1.56 (0.65–3.71)

^*^p-value < 0.05 (significant variation)

### Association of molecular genetic markers with PFS in glioma patients

**Fig. 3:**
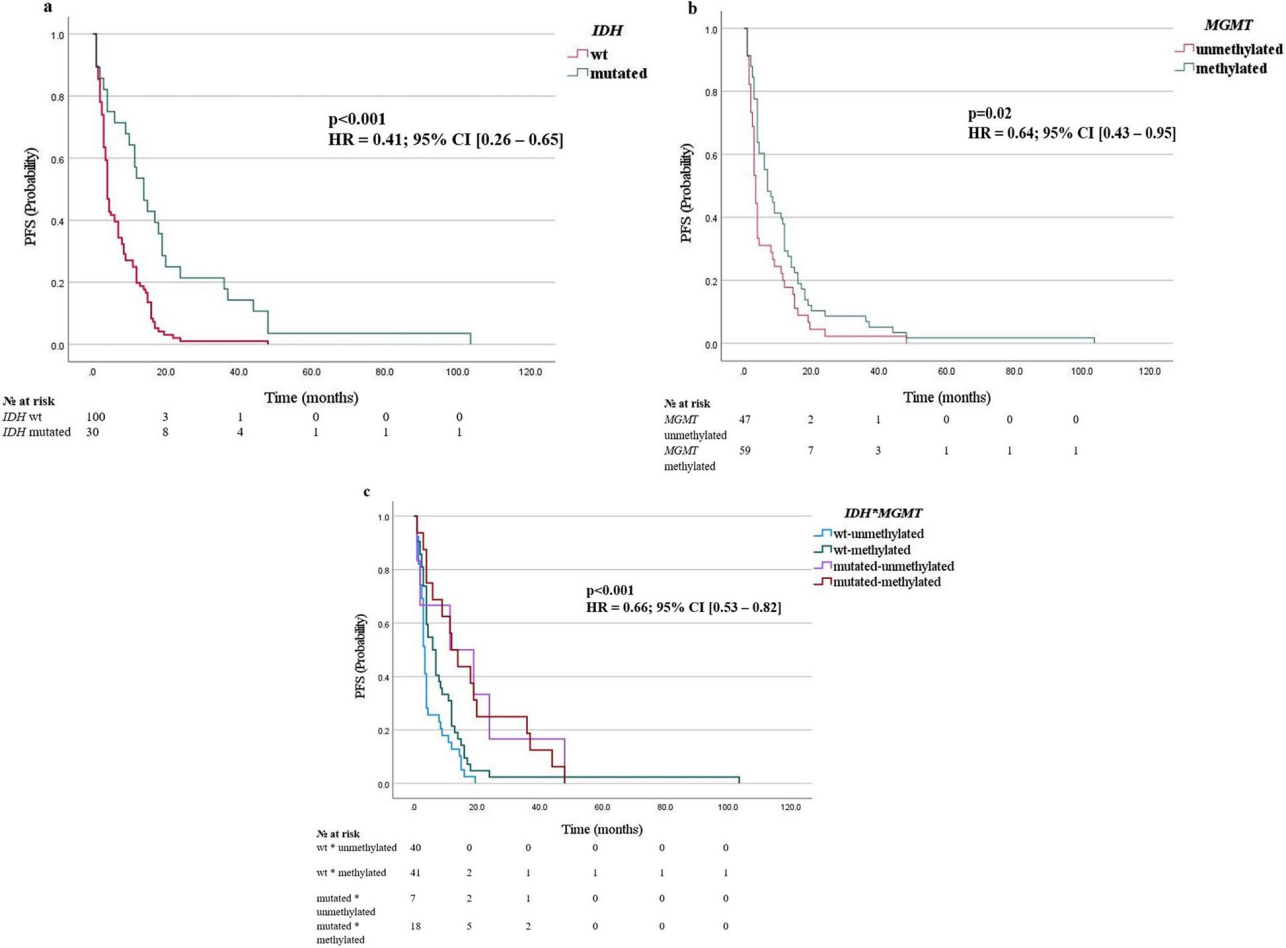
Progression-free survival (PFS) (in months) of glioma patients in **a** mutated and wild-type *IDH*, **b** methylated and unmethylated *MGMT* promoter, and **c** *IDH* mutation × *MGMT* promoter methylation status combinations

Kaplan–Meier survival curves showed that glioma patients with a mutated *IDH* gene have a significantly higher PFS than those with a wild-type gene (Fig. 3a, *χ*^2^ = 16.83, *p* < 0.001; *HR* = 0.41, 95% *CI* [0.26–0.65]). The median survival time for *IDH* mutated patients was 14 months, while it was only 4 months in the *IDH* wild-type group.

Studying the effect of *MGMT* promoter methylation status on PFS, the survival time median was two times higher in patients with a methylated promoter compared to patients with unmethylated ones (7 and 3.5 months, respectively). We have shown that *MGMT* promoter status is significantly associated with PFS in glioma patients (Fig. 3b, *χ*^2^ = 5.37, *p* = 0.02; *HR* = 0.64 95% *CI* [0.43–0.95]).

Next, we assessed the effect of the two studied genetic biomarkers combinations. The presence of the *IDH* mutation with the methylated *MGMT* promoter significantly increased patients’ PFS by more than three times in comparison with patients that have wild-type *IDH* and unmethylated *MGMT* promoter (Fig. 3c, *χ*^2^ = 18.93, *p* < 0.001; *HR* = 0.66, 95% *CI* [0.53–0.82]).

### Association of molecular genetic markers with overall survival (OS)

**Fig. 4:**
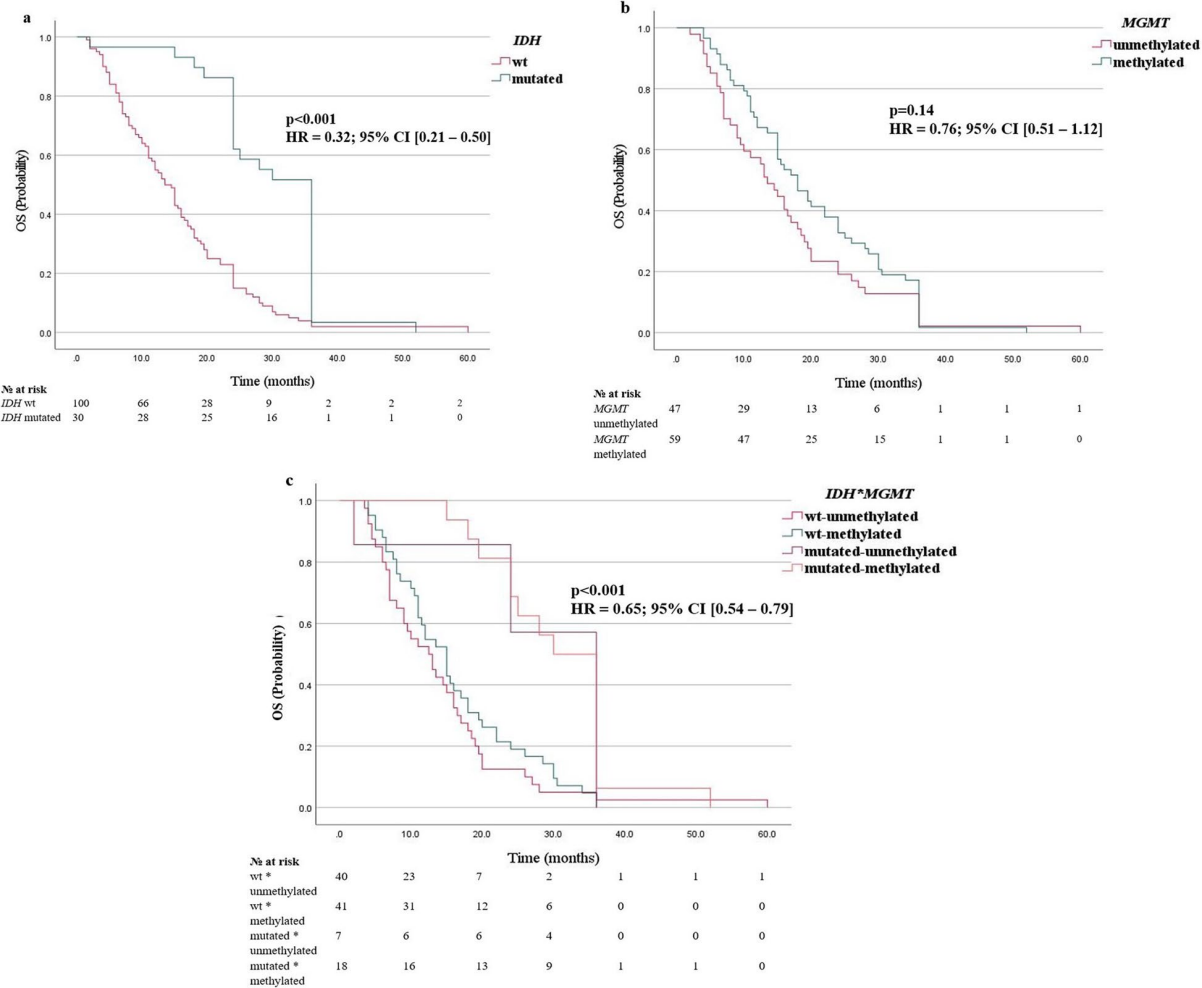
Overall survival (OS) (in months) of glioma patients in **a** mutated and wild-type *IDH*, **b** methylated and unmethylated *MGMT* promoter, and **c** *IDH* mutation × *MGMT* promoter methylation status combinations

According to the Kaplan–Meier test, *IDH* mutations significantly affected the overall survival of glioma patients (*χ*^2^ = 31.12, *p* < 0.001; *HR* = 0.32, 95% *CI* [0.21–0.5]), as shown in Fig. 4a. Patients with mutated *IDH* had about three times higher OS, compared to those with the wild-type variant (36 months and 13.5 months, respectively).

However, although it showed a significant effect on PFS, *MGMT* promoter methylation status did not affect OS of glioma patients (Fig. 4b, *χ*^2^ = 2.2, *p* = 0.14; *HR* = 0.76, 95% *CI* [0.51–1.12]). OS median time for patients with methylated *MGMT* was 18 months, while it was 13.5 months for patients with unmethylated promoter.

The association of genetic markers combination (*IDH* mutations × *MGMT* promoter methylation status) with OS was also assessed. Results showed that intergenic interaction of *IDH* mutations and *MGMT* promoter methylation significantly increases OS by more than two times (Fig. 4c, *χ*^2^ = 26.09, *p* < 0.001; *HR* = 0.65, 95% *CI* [0.54–0.79]). The presence of *IDH* wild-type variant with *MGMT* unmethylated promoter was accompanied by an OS median of 12.5 months, while the carriers of *IDH* mutated × *MGMT* methylated combination had an OS median of 30 months.

## Discussion

During the past years, classification of gliomas has shifted from a basic histological perspective to a molecular biomarkers-dependent classification [2]. The studied genetic markers in gliomas include the following: *IDH* mutations, 1p/19q codeletion, *MGMT* promoter methylation status, epidermal growth factor receptor (*EGFR*) amplification, telomerase reverse transcriptase (*TERT*) promoter mutations, and others [15].

*IDH* mutations are the basic molecular biomarkers in glioma classification, especially in adult-type diffuse gliomas, where *IDH* assessment is required for all cases [22]. According to the results of previous research studies, *IDH1/IDH2* mutations are positive prognostic factors in glioma patients [10, 14, 25]. Furthermore, patients with *IDH*-mutant gliomas showed a longer OS [4, 18] and PFS in different populations [17]. However, such an effect on the survival rate has not been studied before in Russian glioma patients. In this study, the significant association of *IDH* mutations with both OS and PFS was confirmed in a retrospective Russian cohort of patients with different glioma histological subtypes.

The most frequent mutation among glioma patients was *IDH1* (R132H). This is consistent with previous findings, showing that in most cases with *IDH1* mutations, amino acid substitutions are R132H, and this alteration accounts for most of the *IDH* alterations in gliomas. In contrast, the frequency of *IDH2* mutations is generally low (0.3–5.2%) [5, 6].

Among included patients, two male patients, aged 31 and 34 years, had *IDH1* (R132G) and *IDH2* (R172 W), respectively. They also had the same histological glioma subtype (diffuse astrocytoma), and the same therapy tactics (surgical followed by radiotherapy) resulted in long-term stabilization in both of them.

Although it is less studied than *IDH* mutations, methylation of the *MGMT* promoter is already known to increase sensitivity to alkylating agents. Thus, it is used in clinical practice as a positive prognostic factor of different glioma types [1, 26]. The association of this genetic biomarker with different gliomas prognosis was intensively investigated. However, its association with survival rate is less studied. In the current study, the *MGMT* promoter methylation status was found to be significantly associated with PFS, but not OS in Russian glioma patients. In contrast to our result, a previous study found a statistically significant improvement in OS in all patients with methylated *MGMT* [8]. This could be explained by the fact that the mentioned study included only patients with low-grade gliomas (LGG) without considering glioblastoma. On the other hand, Egaña et al. noted that *MGMT* methylation, as an independent factor, has no significant effect on neither PFS nor OS. They suggested that survival variation seemed to be affected by several factors (other than *MGMT* methylation), such as temozolomide cycles or the patient’s general status [7]. Because of the controversial findings concerning the *MGMT* methylation impact on PFS and/or OS, future large-scale studies including all glioma histological subtypes are needed to improve our understanding of the *MGMT* prognostic role in these malignancies.

The interactive effect of *MGMT* with other genetic biomarkers was not considered enough in the literature. In our study, although the frequency of *IDH* mutations was not associated with *MGMT* methylation, the combination of these two genetic biomarkers showed significant associations with both PFS and OS (Figs. 3c and 4c, respectively). The performed Kaplan–Meier analysis suggested that Russian glioma patients with the *IDH* mutated gene and the *MGMT* methylated promoter have a longer PFS and OS rates. Similarly, Chai et al. showed that *MGMT* promoter methylation is significantly associated with increased OS in *IDH*-mutant glioblastoma patients [3]. Another study proved that *IDH1/2* mutations are independent better prognostic factors for survival in glioma patients. It also mentioned that the combination of this factor with *MGMT* methylation points toward strong prognostic implications for longer survival [19].

This study has some limitations, including small sample size (particularly in grade I-II glioma patients’ group) and missing survival data for some patients. In addition, the included Russian patients have been diagnosed with grades I–IV glioma based on the WHO 2016 classification criteria, as the patients were diagnosed between 2018 and 2021.Furthermore, since the studied Russian cohorts were retrospectively recruited in the study, conducting multivariate analysis was not possible. Finally, the absence of validation for methylation results is also considered a limitation.

## Conclusion

To our knowledge, this is the first study to investigate the association of *IDH* and *MGMT* genetic biomarkers with glioma in the Russian population. We have also included all glioma grades, studied both PFS and OS, and analyzed the effect of these two biomarkers as separated factors and their intergenic effect. Results will help in better understanding the genetic basis of glioma pathogenesis. Our findings could be used in future studies to improve glioma prognosis and classification and enable the development of personalized treatment protocols depending on multiple molecular biomarkers.

## Data Availability

No datasets were generated or analysed during the current study.
